# Indian Summer Monsoon Simulations: Usefulness of Increasing Horizontal Resolution, Manual Tuning, and Semi-Automatic Tuning in Reducing Present-Day Model Biases

**DOI:** 10.1038/s41598-018-21865-1

**Published:** 2018-02-23

**Authors:** Abhishek Anand, Saroj K. Mishra, Sandeep Sahany, Mansi Bhowmick, Janmejai Singh Rawat, S. K. Dash

**Affiliations:** Centre for Atmospheric Sciences, IIT Delhi, Delhi, India

## Abstract

Coupled Global Climate Models (CGCMs) of the Coupled Model Intercomparison Project Phase 5 (CMIP5) are unable to resolve the spatial and temporal characteristics of the South Asian Monsoon satisfactorily. A CGCM with the capability to reliably project the global as well as the regional climatic features would be a valuable tool for scientists and policymakers. Analysis of 28 CMIP5 models highlights varying degree of biases in precipitation and 2 m surface air temperature (T2m) over south Asia, and the Community Earth System Model (CESM) developed at the National Center for Atmospheric Research is found to be one of the best performing models. However, like all other CMIP5 models, CESM also has some inherent model biases. Using CESM, it is found that the precipitation and T2M biases reduce with increase in the model horizontal resolution from 2° to 0.5°. Further, a few deep convective parameters in the Zhang-McFarlane convection scheme are tuned for 2° and 1° model resolutions using both manual and semi-automatic model tuning methods. Comparing results from the two tuning methods we find that the performance of the manually tuned model is better than that of the semi-automated one.

## Introduction

Climate simulations performed with coupled global climate models (CGCMs) are widely viewed as the principal scientific basis for developing policies to address potential future global climate change^1^. Intergovernmental Panel on Climate Change (IPCC) has an immense contribution towards the policy-making decisions of many countries and organizations. Hence, accuracy in the long term climatic projections and analysis is of uttermost importance. Scores of modeling institutions^2^ have developed sophisticated CGCMs. No doubt, after decades of development and improvements these models simulate the global climate up to a satisfactory level. Still, these models are plagued by many inherent systematic biases.

There are long standing biases, especially in the tropical regions, that have remained since several generations of models over the decades. Many of these models have participated in the CMIP5^3^ project, with their inherent systematic biases. These biases inherited in the CGCMs pose serious complications in the interpretation of the results generated. Owing to the intricate shape of the Indian land mass and Indian Ocean basin, the region has strong seasonal influence of the Inter Tropical Convergence Zone (ITCZ) variation. These CGCMs are coarser in resolution, so any attempt to oversee their regional imprints suffers from large biases. Attempts have been made to rectify these long-term systematic biases^4–16^ through various methods. Methods that have been used in the past for bias reduction include manual tuning, increasing model resolution^17–19^, and semi-automatic tuning^20^. Each of these tuning methods is robust and self-sufficient. It is important to note that the confidence in the future climatic projections will increase if the models simulate the current climate satisfactorily to start with.

The purpose of this study is to identify the model(s) suitable for global as well as regional (monsoonal) scale simulations with realistic features and explore if some of the widely used model improvement techniques can be applied to the selected model(s) to reduce biases. The existing model biases are computed by inter-comparison with observations and reanalysis datasets^21–23^. In the assessment of biases, this study is confined primarily to the two most important model variables, namely, surface air temperature (T2m) and precipitation. We would like to emphasize that the primary objective of the paper is to check if any of the employed methods (i.e., increased resolution, manual tuning, and semi-automatic tuning) is successful in reducing model biases. The reason for testing these methods is that they are widely used (excepting the semi-automatic tuning which is quite recent and not as widely used) by climate modeling groups worldwide in the process of model improvement. Simple statistical means such as bias correction can easily be used for correcting biases in the mean state as well as the distribution, but that does not lead to model improvement, since it is only a post-processing exercise done to the model outputs. Thus, reducing model biases using statistical techniques is beyond the scope of the current work.

## Results

In the following the monsoon bias in present day models are discussed in section 2.1, followed by sensitivity of model simulations to horizontal resolution presented in section 2.2, response to manual parameter tuning in section 2.3, and finally response to semi-automatic parameter tuning in section 2.4.

### Monsoon Bias in the Present Day Models

In this section monsoon biases in CMIP5 models are described using two statistical measures, namely, the Normalized Root Mean Square Error (NRMSE) and spatial pattern correlation coefficient using Pearson Correlation Coefficient (PCC) calculated over the monsoon region (5°N-38.5°N; 50°E-100° E). These statistical measures are used to assess the accuracy of the model variables with respect to observations. Here observation includes TRMM dataset for precipitation and MERRA dataset for all other variables (see Section 4 for details). For the normalization, Root Mean Square Error (RMSE) is divided by the area averaged observational value. PCC indicates the strength and direction of a linear relationship between two variables. This correlation coefficient is obtained by dividing the covariance of the two variables by the product of their standard deviations. For the calculation of NRMSE and PCC, model results and observations are interpolated bi-linearly to a common grid. In cases where the model is coarser than the observations, which is generally true in our case, the observations are interpolated to the model grid, else vice-versa (done only for MIROC-4h).

**Figure 1:**
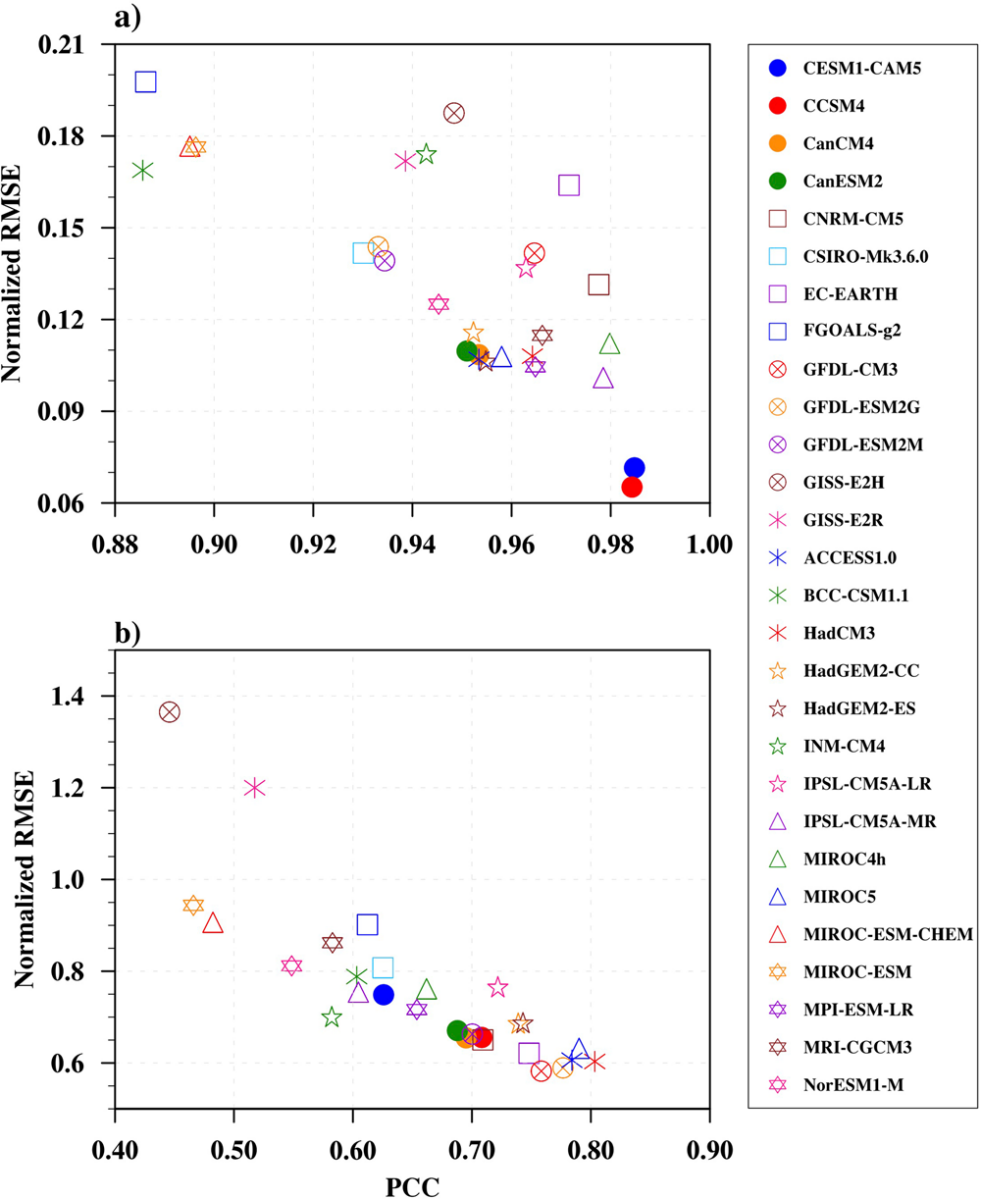
Scatter plot of Pattern Correlation Coefficient (PCC) and normalized RMSE for JJAS mean: (**a**) Surface Air Temperature and (**b**) Precipitation over the monsoon region (5°N-38.5°N; 60°E-100°E). The PCC and normalised RMSE are computed with respect to MERRA and TRMM data for T2m and precipitation, respectively. Figure is created using the NCAR/NCL^52^ software version 6.3.0, https://www.ncl.ncar.edu.

JJAS climatological mean of 31 years (1975–2005) was used from CMIP5 historical datasets. Surface air temperature and precipitation outputs are analyzed for 28 models, through PCC and NRMSE (see Fig. 1). For the selected Monsoon region, the PCC values for T2m and precipitation are positive. 12 and 15 out of the 28 models show PCC values above 0.7 for precipitation and above 0.95 for T2m, respectively. Similarly, around 14 out of 28 models show NRMSE values below 0.7 for precipitation and below 0.12 for T2m.

The CESM1-CAM5 has the highest PCC (0.9848) and low NRMSE (0.0715) value with MERRA T2m. However, FGOALS-g2 has the lowest PCC (0.8862) and highest NRMSE (0.1977) values. Thus, with respect to T2m CESM1-CAM5 is found to have performed better among all the models and FGOALS-g2 stands last. Similarly, HadCM3, MIROC5 and ACCESS1.0 have the PCC above 0.75 and NRMSE around 0.6 with TRMM precipitation, and GISS-E2H has the lowest PCC (0.4458) and highest NRMSE (1.365) among all the 28 models. Therefore HadCM3 is better model among 28 models and GISS-E2H is the less skillful model according to precipitation.

It was found (not shown here) that there is an error of ~10% in the T2m and about 50–100% in JJAS mean precipitation. The margins of error in both the parameters are beyond acceptable range if these models are to be considered for future climate projections.

**Figure 2:**
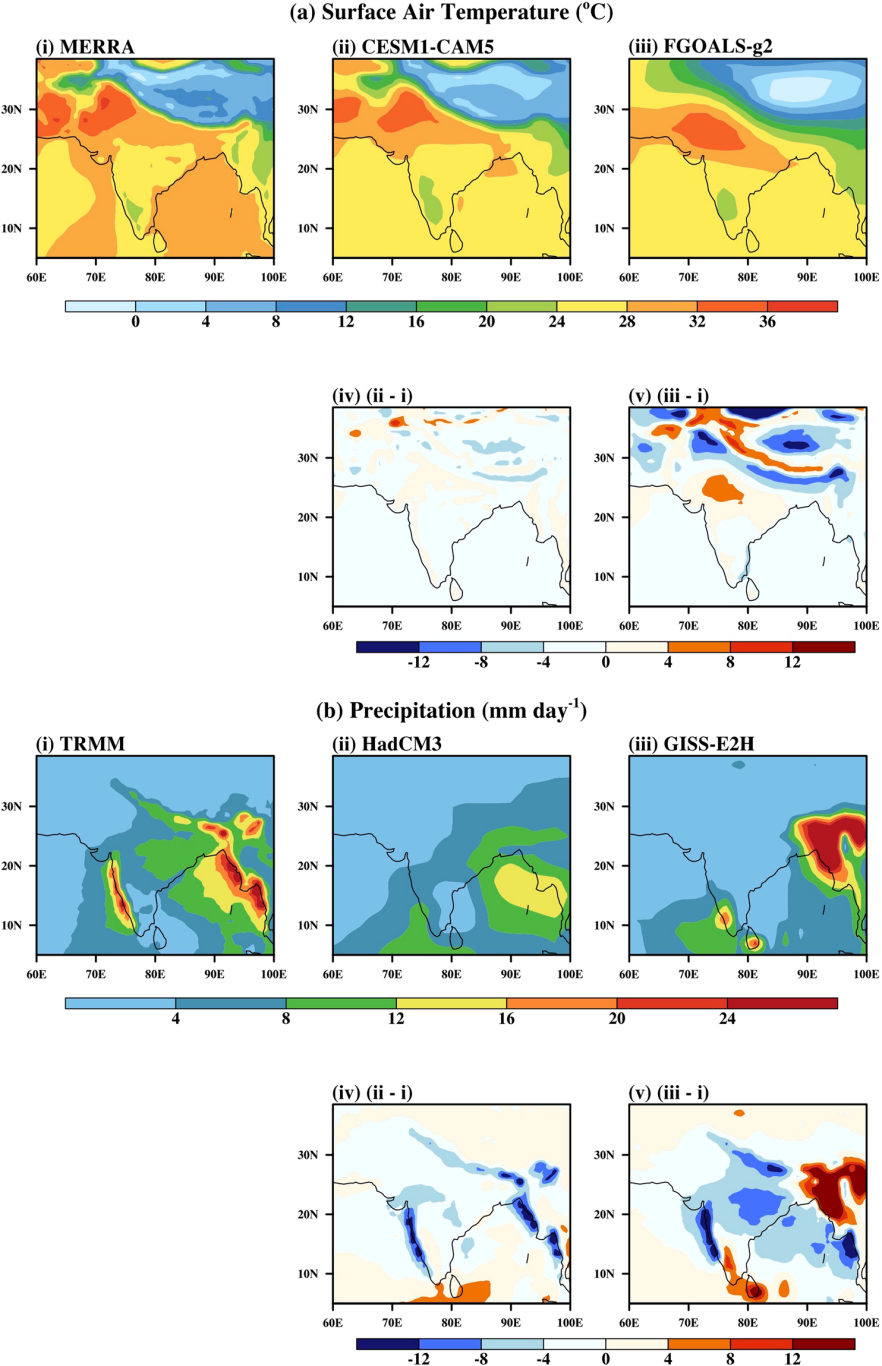
Spatial distribution of JJAS mean: (**a**) Surface Air Temperature in (i) MERRA, (ii) CESM1-CAM5, (iii) FGOALS-g2, (iv) CESM1-CAM5 anomaly and (v) FGOALS-g2 anomaly plots and (**b**) Precipitation in (i) TRMM, (ii) HadCM3, (iii) GISS-E2H, (iv) HadCM3 anomaly, (v) GISS-E2H anomaly over the monsoon region. Figure is created using the NCAR/NCL^52^ software version 6.3.0, URL: https://www.ncl.ncar.edu and Indian boundary data was downloaded from http://www.naturalearthdata.com/downloads in December 2013.

In Fig. 2 spatial patterns for the better and less skillful model from CMIP5 are spatially compared with MERRA reanalysis for T2m and TRMM data for precipitation. Surface air temperature is both underestimated and overestimated over different regions within the monsoon domain in the better model (CESM1-CAM5) as well as the less skillful model (FGOALS-g2). The better model underestimated the high-temperature regions of Arabia, Iran, Arabian Sea, Bay of Bengal, and Indian Ocean and low-temperature regions of Tibet (see Fig. 2a(ii) and (iv)). Few regions like Southern India show an overestimate in the temperature variation, especially over interior Karnataka (see Fig. 2a(iv)). In the less skillful model the center of high temperature over Indian subcontinent is shifted by 5° to the southeast direction (see Fig. 2a(i) and (iii)). By and large, the better model has captured well the broad features of temperature distribution in the area of analysis but the same cannot be said for precipitation (as discussed below).

The better model (HadCM3) underestimates the precipitation over the Western Ghats, Himalayan foothills, and Burmese Mountains (see Fig. 2b(ii) and (iv). The less skillful model (GISS-E2H; (see Fig. 2b(iii) and (v)) underestimates the precipitation over Himalayas, central India, Bay of Bengal and eastern Indian Ocean. It overestimates the precipitation over the southwest tip of India and Burmese Mountains. One of the notable things that can be observed from Fig. 2b is that the model closely follows the demarcation line of high precipitation region to low precipitation region. In a relevant study^24^, the performance of coupled models are analyzed and found large biases in the mean rainfall pattern simulated by the models. They reported that, in general, rainfall over the equatorial Indian Ocean was overestimated, whereas it was underestimated over the Indian sub-continent. They reported that none of the models were able to successfully simulate the major features of Indian monsoon rainfall distribution, such as the maximum over Bay of Bengal and adjoining northeast India and west coast and minimum over NW India and southeast India. The above analysis indicates that model improvement is necessary to generate confidence in future projections. We have selected NCAR CAM5^25^ for further study since it has the best T2m simulations among the 28 CMIP5 models and performs relatively well for precipitation as well.

### Sensitivity to resolution

**Figure 3:**
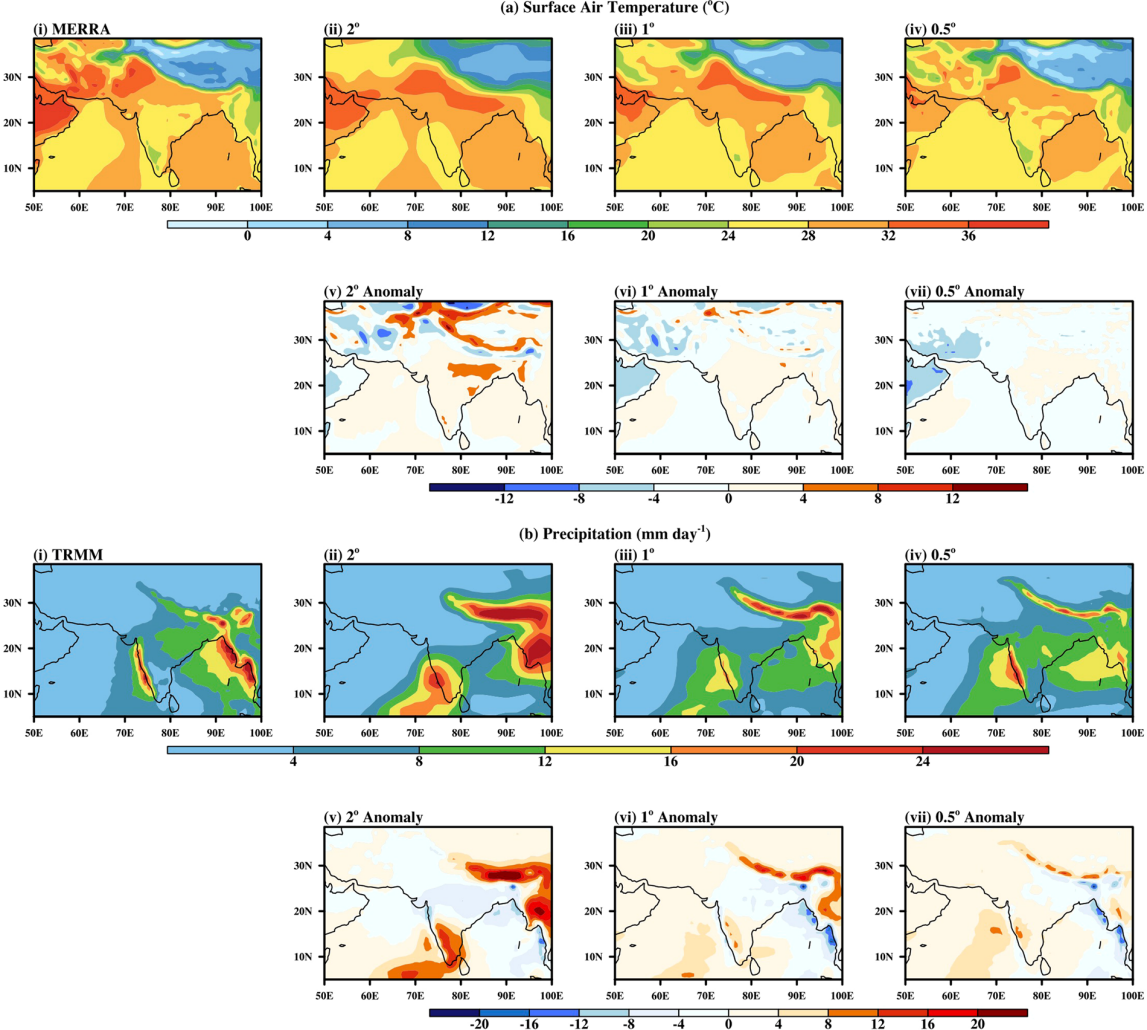
Spatial distribution of JJAS mean: (**a**) Surface Air Temperature in (i) MERRA, (ii) 2°, (iii) 1°, (iv) 0.5° (v) 2° anomaly, (vi) 1° anomaly, (vii) 0.5° anomaly and (**b**) Precipitation in (i) TRMM, (ii) 2°, (iii) 1°, (iv) 0.5° (v) 2° anomaly, (vi) 1° anomaly, (vii) 0.5° anomaly, over the monsoon region. Figure is created using the NCAR/NCL^52^ software version 6.3.0, https://www.ncl.ncar.edu and Indian boundary data was downloaded from http://www.naturalearthdata.com/downloads in December 2013.

**Figure 4:**
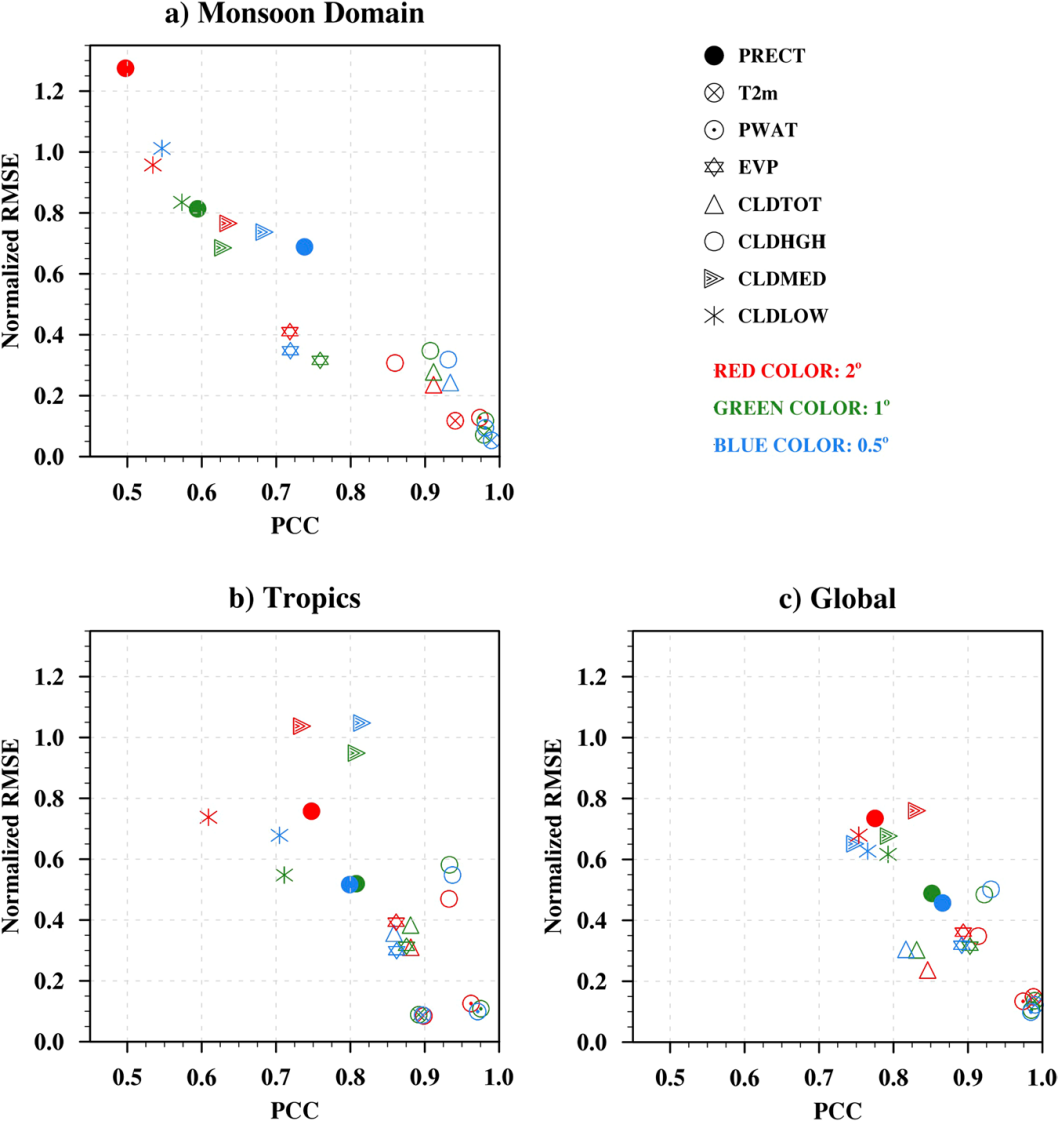
Scatter plot of PCC and Normalized RMSE for (**a**) Monsoon, (**b**) Tropics and (**c**) Global domains. Red, Green and Blue colors represent 2°, 1° and 0.5° resolutions, respectively. We use JJAS mean for monsoon domain and tropical domain, and JJA mean for global domain to correlate with MERRA reanalysis. TRMM data set was used for precipitation in Monsoon and Tropical domains owing to higher accuracy. The various parameters precipitation, T2m, PWAT, EVP, CLDTOT, CLDHGH, CLDMED and CLDLOW represent precipitation, 2 m surface air temperature, precipitable water, surface evaporation, total cloud, high cloud, medium cloud and low cloud, respectively. Figure is created using the NCAR/NCL^52^ software version 6.3.0, https://www.ncl.ncar.edu.

Considering all the models of CMIP5 at different resolutions, most of the models having low resolution show larger bias over land. Thus, the resolution seems to play an important role over land. Therefore, in this section, model sensitivity to the change in the horizontal resolution is studied, for T2m and precipitation parameters. Ensemble of six members (with different initial conditions as discussed in Section 4) for each resolution is considered for the study. Systematic improvement in the simulation pattern (Fig. 3) and reduction in biases (Fig. 4a) for both the parameters is observed with increase in resolution. This improvement in the simulation of surface variables may be attributed to improved representation of orography and coastlines at high horizontal resolution. But the increased resolution does not always lead to an improvement in all the aspects. For example, by comparing the spatial plot of T2m (Fig. 3a) at various model resolutions it is found that the model with higher resolution has a better representation of the spatial features but in terms of magnitude, there is an underestimation over the Arabian Sea region. Some researchers like Bacmeister *et al*.^26^ and others^27–30^ investigated the role of dynamics and physics parameterization in resolution sensitivity using a model with full physics on a standard grid and increasing the resolution of the dynamics. They found that with increase in resolution, regional precipitation features and precipitation intensity distributions improved. A similar result i.e. the improvement in simulations with increasing horizontal resolution is demonstrated in Fig. 3. The impact of an increase in horizontal resolution is seen from 2° to 0.5° on T2m compared with MERRA reanalysis (Fig. 3a(i)) and precipitation compared with TRMM datasets (Fig. 3b(i)). A marked improvement is observed in the T2m (Fig. 3a) from the coarse to fine model resolutions. The spatial patterns of JJAS mean T2m for 2° resolution (Fig. 3a(ii) and (v)) has a large bias over the Indo-Gangetic plains and Tibet over land. Over water bodies, the pattern is similar to the MERRA with few spatial biases over Indian Ocean. In the 1° resolution (Fig. 3a(iii) and (vi)), there is a significant improvement in the T2m with few biases over land mainly due to the enhanced model resolution, which results in a detailed representation of orography, however, no significant improvement is seen over the water bodies. The model with 0.5° resolution (Fig. 3a(iv) and a(vii)) captures well most of the high and low T2m features over Indian sub-continent but underestimates the surface temperature over Arabia, Iran, and oceanic regions.

Largely, increasing the horizontal resolution enhances the spatial precipitation accuracy of the finer model compared to TRMM datasets (Fig. 3b). But again the precipitation is underestimated over the Burmese Mountains and adjoining water bodies. The spatial patterns of JJAS mean precipitation for 2° resolution (Fig. 3b(ii)) has large biases over the Western Ghats, Himalaya, Myanmar and the Indian Ocean regions. These biases decrease significantly for 1° (Fig. 3b(vi)) and 0.5° resolution (Fig. 3b(vii)) owing to improved horizontal model resolution. Still, in all three-model resolutions, the systematic bias is present over the Indian Ocean. These biases show high rainfall over the western Indian Ocean, which is in contrast to the TRMM observations.

Figure 4 describes the PCC and NRMSE variability of model variables like Precipitation (PRECT), Surface Air Temperature at 2m Height (T2m), Precipitable Water (PWAT), Surface Evaporation (EVP), Total Cloud (CLDTOT), High Cloud (CLDHGH), Middle Cloud (CLDMED) and Low Cloud (CLDLOW) in three domains, namely, Monsoon (50°E:100°E, 5°N:38.5°N), Tropics (0°E:360°E, 30°S:30°N), and Global (0°E-360°E, 90°S-90°N). The statistical measures of model variables are demonstrated for three horizontal resolutions, 2° (red color), 1° (green color), and 0.5° (blue color). Precipitation in the Monsoon and Tropical domains have been compared with 10 year TRMM data as it has higher grid resolution, while 10 years of MERRA reanalysis was used for comparison in the Global domain. Precipitation has higher pattern correlation and low NRMSE in global domain compared to the Monsoon and Tropical domains, with higher resolution. Being a matter of surprise, precipitation in the tropical domain has better performance for 1° resolution. T2m in the Monsoon domain (Fig. 4(a)) can be identified distinctly, with a higher resolution mostly having higher PCC and lower NRMSE values. Similar features are seen in Fig. 4(c) but with partial overlapping. In Fig. 4(b), over the tropical region, 2° resolution model has better correlation, closely followed by 0.5° model resolution, with 1° model ending last. PWAT in all three domains (Fig. 4(a–c)) follow the normal trend of improvement with increasing resolution, but between 1° and 0.5° the improvement is very small. For EVP, the above trend is not followed where 1° takes the lead followed by 0.5° model resolution and 2° model is the last one in all the three domains. CLDTOT and CLDHGH have high PCC and low NRMSE for 0.5° in Monsoon Domain (Fig. 4(a)). In the Tropical domain, a similar pattern is followed. On the other hand, the Global domain indicates that 2° model has the best PCC and 0.5° has the worst values of PCC and NRMSE for CLDTOT. In Tropics and Global domain, 0.5° model resolution has better values of PCC and NRMSE followed by 1° and 2°, respectively. For CLDMED variable in the Monsoon and Tropics, there is an improvement in the value of PCC with an increase in resolution. In the Global domain, depreciation in PCC but improvement in NRMSE is observed with increase in resolution. For CLDLOW variable, in Fig. 4(a–c), the 1° model resolution has better values of PCC and NRMSE followed by 0.5° and 2°, respectively.

In most of the cases the increase in model resolution helps in better representation of the model variables, especially for T2m, precipitable water and precipitation (not in tropics) in all the three study domains namely Monsoon, Tropics, and Global.

### Response to Manual Parameter Tuning

Tunable parameters^31,32^ are often used in the climate model parameterization schemes^33^. The aim of the current model tuning exercise is to quantify and optimize the model for deep convective parameterization scheme. In CAM5, the deep convective process is parameterized by the Zhang and McFarlane deep convection scheme, which was originally developed by Zhang and McFarlane^34^ (ZM) with additional modifications^35–37^. ZM convective scheme in CAM5 only represents convective precipitation associated with updrafts and saturated downdrafts. Several key parameters related to the process of updrafts, downdrafts, cloud-rain conversion and convective available potential energy (CAPE) are important^38^ in the ZM deep convection scheme^34^ but the range of values for these parameters are uncertain.

**Figure 5:**
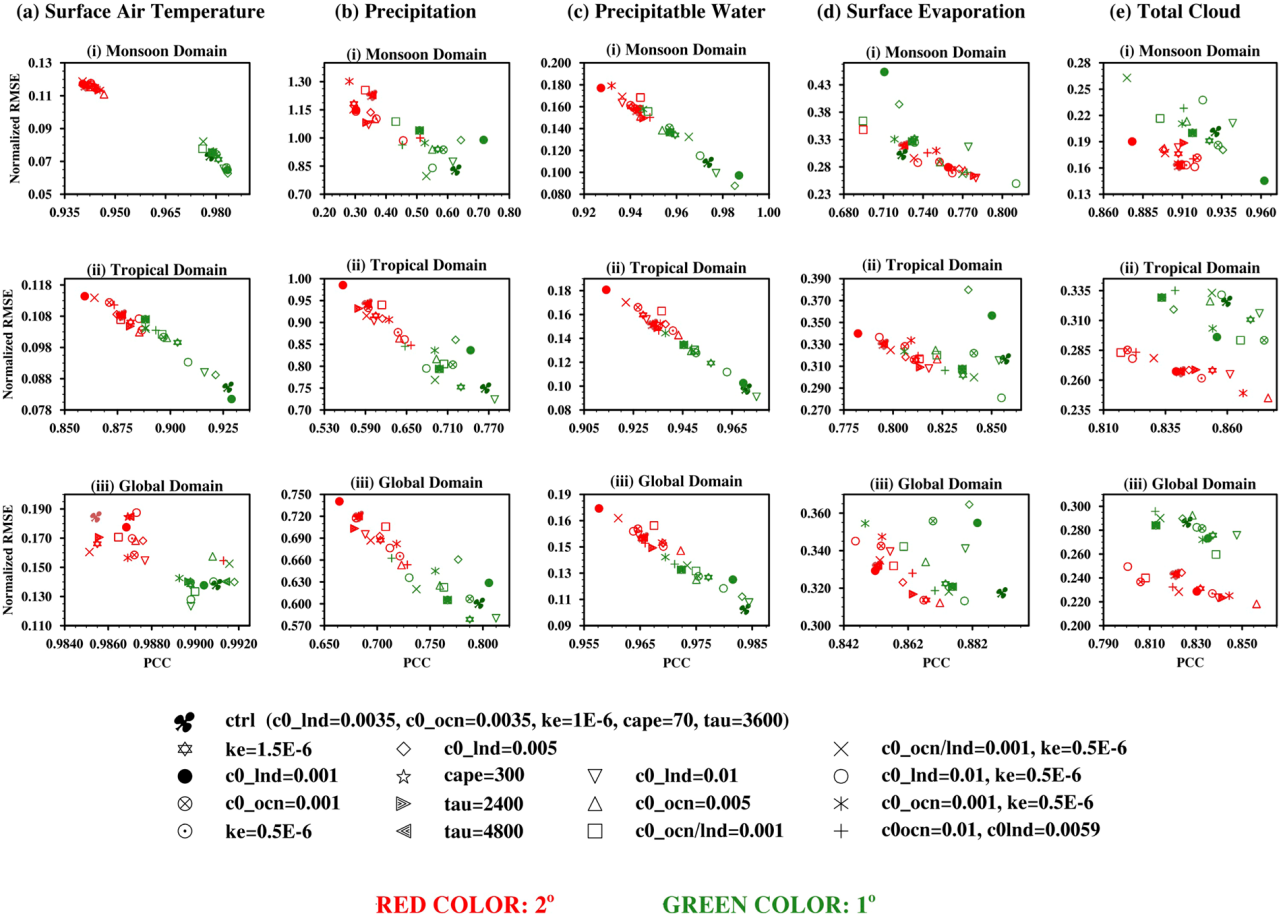
The scatter plot of PCC and Normalized RMSE is shown for five variables: (**a**) Surface Air Temperature, (**b**) Precipitation, (**c**) Precipitable Water (**d**) Surface Evaporation and (**e**) Total Cloud for three different domains: (i) Monsoon, (ii) Tropical and (iii) Global domains. Red and Green colors represent 2° and 1° resolutions, respectively. We use JJAS mean for monsoon domain and tropical domain, and JJA mean for global domain to correlate with MERRA reanalysis. TRMM dataset was used for precipitation in Monsoon and Tropical domains owing to higher accuracy. The various parameters represented here are from the Zhang-McFarlane convection scheme. Figure is created using the NCAR/NCL^52^ software version 6.3.0, https://www.ncl.ncar.edu.

A number of simulations with various combinations of values for tunable parameters were performed for a short period of 6 months (starting April 1^st^). More than 60 simulations for each of 2° and 1° model resolution were performed for different parameters listed in Table 1. Here we have summarized results of best 15 simulations for each of the model resolutions. Figure 5 demonstrates NRMSE along with PCC for Indian Monsoon region, Tropics and Global domains. The five variables that are represented here are Surface Air Temperature, Precipitation, Precipitable Water, Surface Evaporation and Total Cloud. Discussion of various parameters for the above-mentioned three regions has been done below.

**Table 1:** List of parameters those have been modified or tuned.

Parameter	Control Value	Permitted Range	Values Used	Description
C_0__lnd	0.0035	0.001–0.045	0.001, 0.005, 0.01, 0.0059, 0.0035	Deep convective precipitation efficiency over land (m^−1^)
C_0__ocn	0.0035	0.001–0.045	0.001, 0.005, 0.01, 0.0035	Deep convective precipitation efficiency over ocean (m^−1^)
K_e_	1.0E-06	0.5E-6-10E-6	0.5E-6, 1.0E-06, 1.5E-06	Evaporation efficiency of precipitation ((kg m^−2^ s^−1^)^−1/2^ s^−1^)
CAPE	70	20–300	70, 300	Threshold value of CAPE for deep convection (m^−2^ s^−2^)
Ţ (tau)	3600	1800–28800	2400, 4800	CAPE consumption time scale (s)

The T2m as seen in Fig. 5a(i) shows very clear and distinct model variability for both the resolutions, having a clear demarcation in the monsoon domain. Model tuning does not lead to much improvement in PCC of the T2m in the Tropical domain (Fig. 5a(ii)). One can see that the best model configuration in the 1° resolution turns out to be the worst 2° model configuration. In the Global domain (Fig. 5a(iii)), one of the 2 ° model configuration (c0_ocn = 0.01, c0_lnd = 0.0059) has high PCC and is in the range of 1° model simulations. Here the range of PCC values for both the resolutions are very close. For the T2m, the model has higher pattern correlation, especially on the global scale. After model tuning, T2m shows improvement in the range 2–16% for 2° resolution and 1–8% for 1° resolution from the respective control simulations.

For Precipitation (Fig. 5b(i–iii)), the impact of an increase in resolution is seen as an improvement in precipitation values. The model configuration (c0_ocn = 0.01, c0_lnd = 0.0059) has the best value of PCC and NRMSE for 2° model and worst value of PCC and NRMSE for 1°. Thus, one may conclude that a model setup that works for the 2° resolution may respond differently to 1° model resolution. The model CTL(s) with 1° resolution is among the top five best configurations, while last five for the 2° resolution. In general, PCC values increase and NRMSE values decrease as the domain size is increased from Monsoon to Tropical to Global. The model precipitation has improved in the range, 10–25% for 2° resolution and in the range of 4–9% for 1° resolution, after model tuning.

Impact of resolutions is also seen on Precipitable Water from Fig. 5c(i–iii). 1° resolution CTL simulation, similar to precipitation, has values of PCC and NRMSE in top four. Some of the model configurations of 2° resolutions show better performance than few of the model configuration of 1° resolution. For Precipitable Water, the improvement after model tuning for 2° resolution is in the range of 4–8% and for the 1° resolution, it is in the range 1–4%.

There is clear improvement in surface evaporation (EVP) values (see Fig. 5d(i–iii)) with increase in horizontal resolution. However, few model configurations (c0_ocn = 0.005 and c0_ocn = 0.001; ke = 0.5E-6) with 2^o^ resolution perform much better than 1^o^ model configurations. In the 1^o^ resolution setup of global domain, CTL has the best configuration, and among top five in the rest of the two (tropical and monsoon domains). Evaporation parameter has improved in the range 7–16% for 2^o^ resolution and 1–7% for 1^o^ resolution after model tuning.

Similar to Evaporation, the CLDTOT parameter (Fig. 5e(i–iii), it is found that 2^o^ model configurations perform much better than 1^o^ model configurations in all domains. In the Monsoon domain, coarse model configurations have the best NRMSE values and comparable PCC values. The 2^o^ model configuration (c0_ocn = 0.005) performs best for CLDTOT. After model tuning, CLDTOT shows an improvement in the range 7–16% for 2^o^ resolution and 3–10% in 1^o^ resolution.

**Figure 6:**
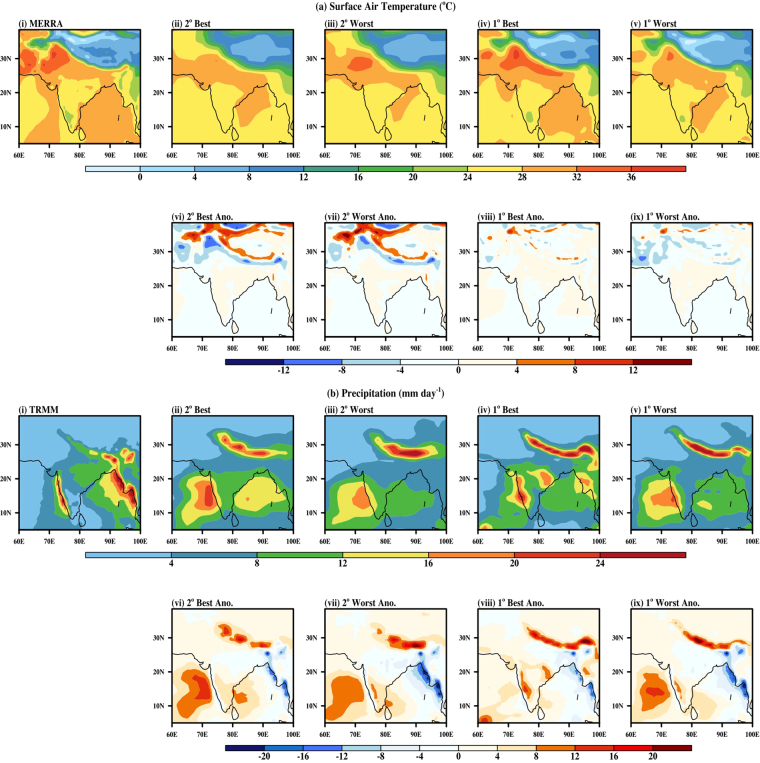
Spatial pattern of JJAS mean: (**a**) Surface Air Temperature in (i) MERRA, (ii) 2° Best, (iii) 2° Worst, (iv) 1° Best anomaly, (v) 1° Worst anomaly and (**b**) Precipitation for (i) TRMM, (ii) 2° Best, (iii) 2° Worst, (iv) 1° Best anomaly, (v) 1° Worst anomaly, over the monsoon domain. Figure is created using the NCAR/NCL^52^ software version 6.3.0, https://www.ncl.ncar.edu and Indian boundary data was downloaded from http://www.naturalearthdata.com/downloads in December 2013.

The spatial plot representing T2m and precipitation can be seen in Fig. 6. The best and the worst model configuration based on NRMSE and PCC, for both resolutions, are compared with MERRA and TRMM datasets. Figure 6a(ii,vi) show that the best model configuration at 2^o^, in general, underestimates the T2m by 2 °C but simulated well the observed broad features of MERRA data, especially over land and Bay of Bengal. Similarly, the 1^o^ model has underestimated T2m over Arabia and Iran (see Fig. 6a(viii)). It has overestimation over Gangetic plains, Arabian Sea and the Indian Ocean. It has very well captured the summer heat low over the western India and Pakistan and over south India (Fig. 6a(iv)).

The precipitation variation simulated by the models and obtained from TRMM dataset is depicted in Fig. 6b (i–v) and anomalies are shown in Fig. 6b(vi–ix). All the models show overestimation in simulating precipitation. The 1° resolution best model configuration broadly captures the prime features of Indian monsoon precipitation, although with few biases. These biases include overestimation of precipitation over the Himalayas, Western Ghats, Central India and Eastern Indian Ocean. The model underestimates the precipitation over Myanmar coastline.

Thus, it is seen that the model tuning process implemented on 2° resolution yields significant improvement (on an average 16%) in all the five parameters, for the three domains. The 1° model resolution shows only mild improvement of 8% at the maximum even after rigorous manual parameter tuning. This may be due to the fact that CAM5 used here was developed and already tuned for 1° resolution. Here it may be noted that the 5 parameters chosen (Table 1) are based on existing literature as well as our own experience. The reported range of percentage improvement may slightly vary by either having a smaller subset or a different set of parameters. It is also seen that the 2° model could be made as good as 1° one. Considering the example from Fig. 5b(iii), the model configuration (c0_ocn = 0.01 and c0_lnd = 0.0059) has best values for 2° model resolution and has even higher values from its 1° model resolution counterpart. It is better than at least 5 model configurations of 1° model. So if this configuration of 2° model resolution is fine-tuned further it could be further improved. If this 2° model configuration is made as default it could save huge computing power and resources. Therefore, it can be concluded that the coarse grid models can be suitably tuned to match most of the results of fine grid models.

### Response to Semi-Automatic Parameter Optimization Method

Traditionally, model development has prospered and progressed through time intensive manual tuning methods, involving comprehensive comparison of model parameters with observations. With the advent of various optimization methods, attempts have been made to automate these time intensive processes. There are several algorithms such as Simulated Stochastic Approximation Annealing (SSAA) method^39^ and Multi-Objective Particle Swarm Optimization (MOPSO) method^40^, to name a few. These, being modern and effective, require more computational simulations to reach the optimal solutions^41^.

The experimental setup used here utilizes Downhill Simplex method for optimization of the convective parameters, proposed by Nelder and Meade^42^ in 1965. This is a heuristic method that does not require derivative of the function but only function evaluation for its operation. The Downhill Simplex is a fast converging algorithm when the parametric space is not high dimensional. However, it is a local optimization algorithm, not aiming to find the global optimal solution. Moreover, the algorithm has convergence issues when the simplex becomes ill conditioned. A three-step method is used to reduce the number of parameters for analysis, which favours the fast convergence of Downhill Simplex method. Also, since it performs the best optimization under local conditions and owing to computational costs, the experiment is designed and simulated only over the domain extending from 0–40°N and 60–100°E. Results of the study are summarized for Indian landmass only. The details of the method adopted are described in Methodology section at the end.

**Figure 7:**
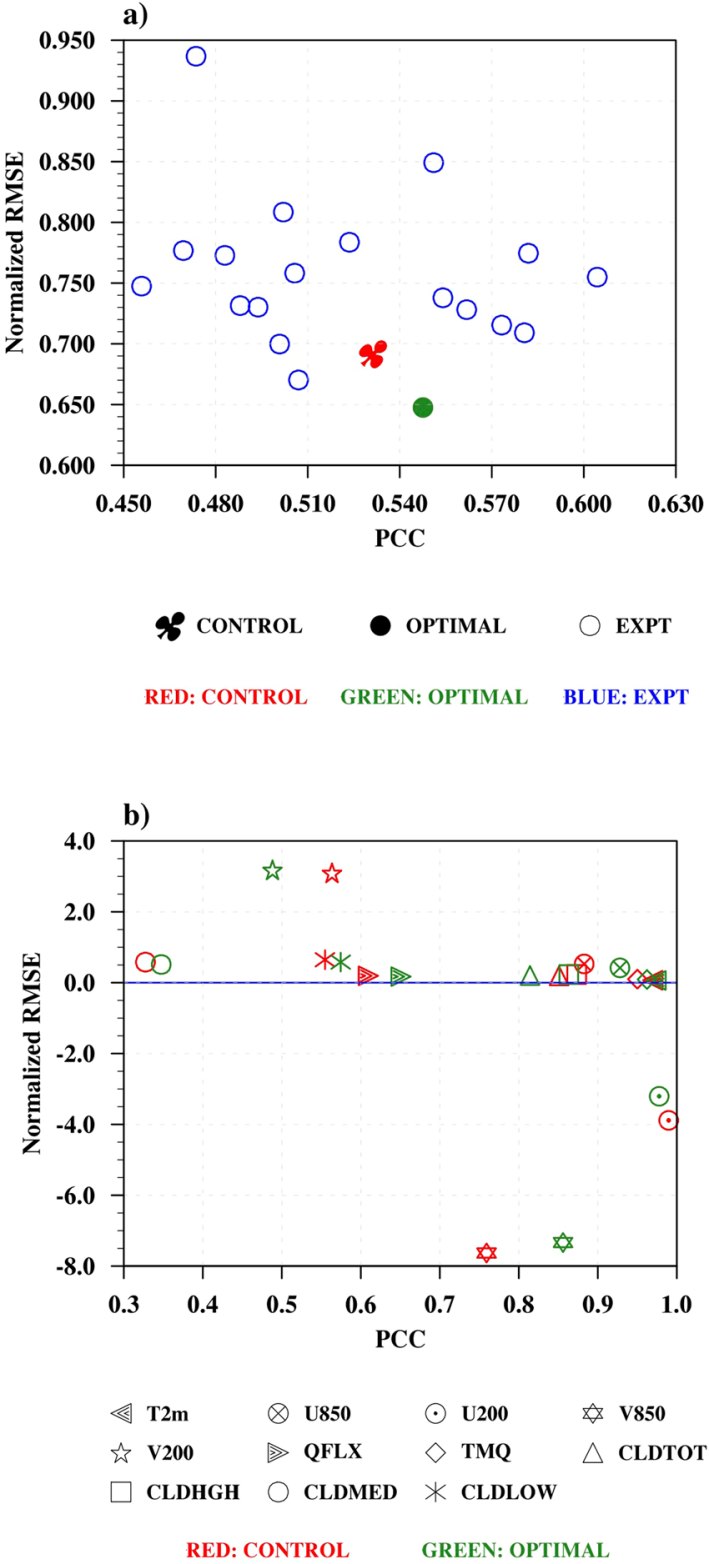
(**a**) Scatter plot of PCC and Normalized RMSE for control, optimal and experimental simulations over Indian landmass. Model simulation for precipitation is compared to IMD observational dataset. Red, Green and Blue colors represent control, optimal and experimental simulations. (**b**) Scatter plot of PCC and Normalized RMSE for control and optimal simulations over Indian land for different variables. Model simulations are compared to MERRA dataset. Various parameters T2m, U850, U200, V850, V200, EVP, PWAT, CLDTOT, CLDHGH, CLDMED and CLDLOW represent 2m surface air temperature, zonal wind at 850 hPa, zonal wind at 200 hPa, meridional wind at 850 hPa, meridional wind at 200 hPa, surface evaporation, precipitable water, total cloud, high cloud, medium cloud and low cloud, respectively. Figure is created using the NCAR/NCL^52^ software version 6.3.0, https://www.ncl.ncar.edu.

A control (CTL) experiment with April initial condition was initiated and 19 successive simulations were performed by Downhill simplex method to reach the optimal simulation. In Fig. 7(a), the NRMSE along with PCC is shown for precipitation. In this figure, the successive simulations are depicted as blue circles, CTL experiment is denoted by red spade, and the optimized (OPT) experiment with a filled green circle. It is seen that the optimized value has the best NRMSE amongst all the experiments performed, but it is not having the best PCC value.

In Fig. 7(b), the PCC and NRMSE are shown for T2m, U850, U200, V850, V200, EVP, PWAT, CLDTOT, CLDHGH, CLDMED, and CLDLOW over Indian land. The variables from CTL simulation are shown in red, whereas, OPT variables are shown in green. In most of the OPT experiment cases e.g., T2m, U850, V850, EVP, PWAT, CLDMED, and CLDLOW, improvement in NRMSE and PCC by 2–6% is seen, whereas, U200, V200, CLDTOT and CLDHGH have better values for CTL experiment.

**Figure 8:**
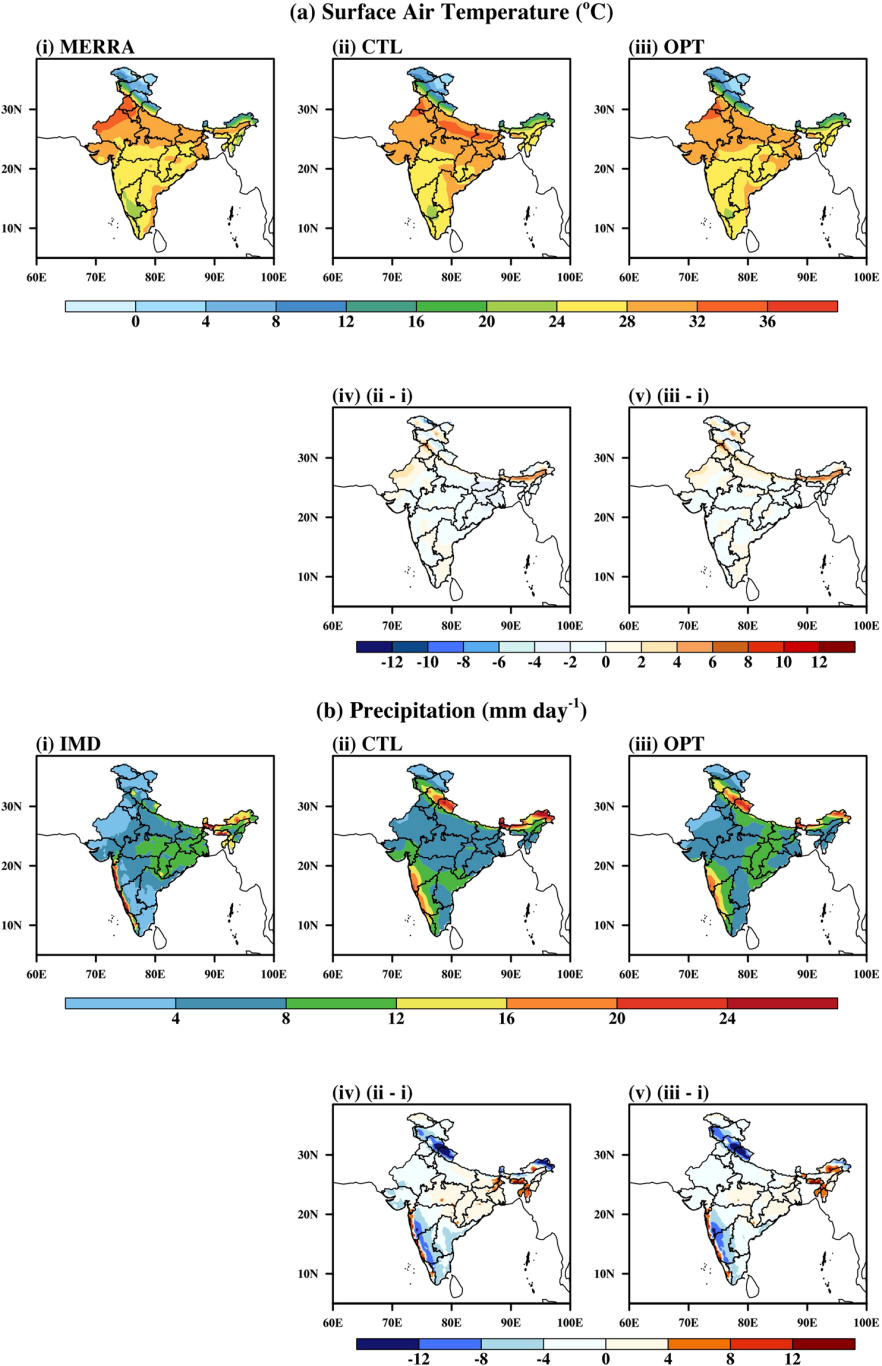
Spatial pattern of JJAS mean: (**a**) Surface Air Temperature from (i) MERRA, (ii) CTL, (iii) OPT, (iv) CTL anomaly, (v) OPT anomaly and (**b**) Precipitation from (i) IMD, (ii) CTL, (iii) OPT (iv) CTL anomaly, (v) OPT anomaly over Indian land. CTL is control model simulation and OPT is optimal model simulation. Figure is created using the NCAR/NCL^52^ software version 6.3.0, https://www.ncl.ncar.edu and Indian boundary data was downloaded from http://www.naturalearthdata.com/downloads in December 2013.

Figure 8(a) represents the spatial pattern of the JJAS mean T2m for CTL and OPT, compared with those in MERRA dataset. The spatial pattern of the JJAS mean precipitation in CTL and OPT is compared with IMD data in Fig. 8(b). The CTL and OPT do not have much difference in T2m. Similarly, OPT is not better than CTL when compared for precipitation. On comparing with IMD values, OPT and CTL configuration mostly overestimated the precipitation, especially over mountainous regions.

## Discussion

Although most of the GCMs^43^ are developed with an objective to accurately simulate the global climate, simulating the regional climate of the corresponding model development centers is often the highest priority. Also, it is not practical for every country to have enough resources for developing their own climate model. Large biases in climate simulations over the Indian region leads to low confidence in the climate change projections and the subsequent impact assessment studies. Also, such models cannot be used for process understanding, hypothesis testing, and future projections due to the high degree of non-linearity in the system. However, building a model with zero bias may not be practically feasible. Thus, instead of hoping for a model with zero bias in all the relevant climatic parameters, we should make efforts to reduce the bias to the maximum extent possible. Seasonal mean T2m and precipitation are the most important parameters that are required in several impact assessment studies and hence have been used in this study.

The model tuning through manual methods is exhaustive and time-consuming. Yet, it provides better model configuration for simulation of regional climate. The results from this study show that increase in horizontal resolution alone will not improve the simulation effectively unless the model is simultaneously tuned. On the other hand, not much can be achieved simply by manually tuning the model. We show that automatic parameter tuning is not that effective, specifically in the context of Indian summer monsoon simulations. Therefore, it is suggested that both manual and automated tuning should go hand in hand. The existing CGCMs are developed at reputed institutions keeping the characteristics of climates of certain specific regions in mind. Therefore, in order to simulate the spatial and temporal characteristics of T2m and precipitation close to their observed values, it is paramount to adopt a combination of manual and automated tuning methods simultaneously along with enhancing the resolution of the model. Efforts are going on in this direction by identifying the sources of biases at each important stages of model tuning.

## Methodology

The study in Section 2.1 uses 28 models from the CMIP phase 5 project (list in the legend of Fig. 1), downloaded from http://cmip-pcmdi.llnl.gov/cmip5/availability.html. Details of the models and data used are available at http://cmip-pcmdi.llnl.gov/cmip5/guide_to_cmip5.html. Standalone Community Atmosphere Model version 5.1 (CAM 5.1)^44^ is used for rest of the study, which is the atmospheric component of Community Earth System Model (CESM). CESM is a coupled climate model, developed at the National Center for Atmospheric Research (NCAR). Details of the NCAR CAM model are available at http://www.cesm.ucar.edu/models/cesm1.0/cam/docs/description/cam5_desc.pdf.

CAM5 simulations are carried out using finite volume (FV) dynamical core and CAM4 physics. Shallow and deep convections are parameterized using the methods described by Park and Bretherton (2009)^45^ and Zhang and McFarlane (1995)^34^, respectively. The model simulations are performed at three different resolutions 1.9° × 2.5°, 0.9° × 1.25° and 0.45° × 0.63° using FV dynamical core referred as 2°, 1° and 0.5° in the text. The six members of each of the ensembles are initialized with initial conditions at 6-hour intervals starting from 1800 UTC 31 March 2000. The initial conditions at 6-hour intervals are produced from a separate model run initialized at 0000 UTC 01 January 2000. The SSTs are prescribed using 20-year climatological (1982 to 2001) mean with monthly variations, downloaded from the NCAR website. Model is integrated for a period of 6 months. The first 2 months are discarded as spin-up. Model performance is evaluated against observations from Tropical Rain Measuring Mission (TRMM)^46^, product 3B43 and Modern-Era Retrospective analysis for Research and Applications (MERRA)^47^ reanalysis data set. The CAM5 experiments were also conducted using different parameter combinations for manually tuned Zhang and McFarlane scheme for the same 6-month period. The manually tuned simulations with changes in the deep convective scheme were performed for 2^o^ and 1^o^ model resolutions. Due to limited computing resources the automated model tuning method (Downhill simplex method) was performed only at 1^o^ model resolution and with India Meteorological Department (IMD)^48^ land only data for precipitation.

Automated model tuning methodology involves three steps, which makes it different from other automation methods. In the first step, the Morris One At a Time (MOAT) ^49,50^, a global sensitivity analysis method, is used to find parameters sensitive to the region. In the second step, suitable initial conditions are found for the incitation of the algorithm. Another global method by Sobol^51^ is then used to validate the results of the Morris method. In the third and final step, the optimal values of sensitive parameters for the model are estimated using the Downhill Simplex algorithm by iteratively solving the optimization problem. All Figures are created using the NCAR/NCL^52^ software version 6.3.0, https://www.ncl.ncar.edu and Indian boundary data was downloaded from http://www.naturalearthdata.com/downloads in December 2013.
